# Direct Evidence for Using *Coriandrum sativum* var. *microcarpum* Essential Oil to Ameliorate Scopolamine-Induced Memory Impairment and Brain Oxidative Stress in the Zebrafish Model

**DOI:** 10.3390/antiox12081534

**Published:** 2023-07-31

**Authors:** Ion Brinza, Razvan Stefan Boiangiu, Oana Cioanca, Monica Hancianu, Gabriela Dumitru, Lucian Hritcu, Gheorghe-Ciprian Birsan, Elena Todirascu-Ciornea

**Affiliations:** 1 Department of Biology, Faculty of Biology, Alexandru Ioan Cuza University of Iasi, 700506 Iasi, Romaniarazvan.boiangiu@uaic.ro (R.S.B.);; 2 Department of Pharmacognosy, Faculty of Pharmacy, Grigore T Popa University of Medicine and Pharmacy Iasi, 700115 Iasi, Romania

**Keywords:** *Coriandrum sativum*, volatile oil, *Danio rerio*, memory, dementia, oxidative stress, Alzheimer’s disease, scopolamine

## Abstract

Essential oil from *Coriandrum sativum* has been demonstrated to provide various pharmacological properties, such as antioxidant, antimicrobial, antibacterial, antifungal, antidiabetic, anticonvulsive, anxiolytic-antidepressant, and anti-aging properties. This study investigated the mechanism of *Coriandrum sativum* var. *microcarpum* essential oil (CSEO, 25, 150, and 300 μL/L) and cognitive impairment and brain oxidative stress in a scopolamine (SCOP, 100 μM) zebrafish model (*Danio rerio*) of cognitive impairment. Spatial memory, response to novelty, and recognition memory were assessed using the Y-maze test and the novel object recognition test (NOR), while anxiety-like behavior was investigated using the novel tank diving test (NTT). The cholinergic system activity and brain oxidative stress were also evaluated. CSEO was administered to zebrafish once a day for 21 days, while SCOP and galantamine (GAL, 1 mg/L) were delivered 30 min before behavioral testing and euthanasia. Our data revealed that SCOP induced memory dysfunction and anxiety-like behavior, while CSEO improved memory performance, as evidenced by behavioral tasks. Moreover, CSEO attenuated SCOP-induced brain oxidative stress and decreased acetylcholinesterase (AChE) activity. The results demonstrated the potential use of the CSEO in providing beneficial effects by reducing memory deficits and brain oxidative stress involved in the genesis of a dementia state.

## 1. Introduction

Alzheimer’s disease (AD) is a neurodegenerative condition, classified as the most common form of dementia, which occurs especially in elderly people. The distinctive and emerging pathological features of the disease are the presence of β-amyloid plaques and neurofibrillary tangles (NFTs) in the brain [1]. AD is clinically characterized by the progressive deterioration of cognitive functions and by the appearance of states of anxiety, apathy, and delusions. Previous research has highlighted the involvement of various brain regions and neurotransmitter systems in anxiety and memory processes, including the amygdala, hippocampus, prefrontal cortex, and cholinergic systems [2,3]. Exploring the possible interactions between *Coriandrum sativum* var. *microcarpum* essential oil (CSEO) and these neural substrates could provide insights into the molecular mechanisms underlying the observed behavioral and antioxidant effects. For instance, investigating the influence of CSEO on neurotransmitter levels, receptor expression, or signaling pathways associated with oxidative stress and cognition could help elucidate the specific targets through which CSEO exerts its effects [4,5]. It has been documented that CSEO is a rich source of bioactive compounds (linalool, γ-terpinene, α-pinene, pinocarvone, carvone, and β-ocimene) with cognitive-enhancing and antioxidant activities in the amyloid β(1–42) rat model of AD [6]. The evolution of irreversible and drastic changes in behavior and personality has been described in AD patients [7,8]. Concomitantly, a degradation of acetylcholine (ACh) and butyrylcholine (BCh) levels persists in the brain of AD patients. Decreased levels of ACh and BCh are thought to be due to the increased activity of acetylcholinesterase (AChE) and butyrylcholinesterase (BChE), which also increase the rate of aggregate Aβ peptide aggregation [9,10]. In addition, Aβ-mediated oxidative stress results in mitochondrial dysfunction, loss of proteostasis, impaired glucose metabolism, altered synaptic plasticity, altered signal transduction, induction of neuroinflammation, and progressive loss of cholinergic neurons [11]. All these manifestations impose a heavy burden on families and society [12]. It has been documented that AD is the sixth leading cause of death in the United States. The total cost of treating individuals with AD and associated dementia is projected to increase from $321 billion in 2022 to over $1 trillion by 2050. The indirect costs associated with chronic illness, such as reduced productivity, diminished quality of life, and increasing dependence on unpaid care provided by family members, contribute to the overall economic and societal consequences of these conditions [13].

Biomedical research depends on the use of laboratory animals to better understand the etiology, pathogenesis, and evolution of human diseases at the cellular and molecular level, and to provide diverse systems for the creation, selection, development, and testing of new therapies [14]. Moreover, animal models ensure the consistency and validity of in vitro research results without subjecting humans to possible risks [15]. The zebrafish (*Danio rerio*) is a small, freshwater teleost that belongs to the family *Cyprinidae* [16]. Since 1980, this fish has been used as a model organism in multiple biomedical fields such as immunobiology, developmental biology, toxicology, neurophysiology, oncology, biomedicine, genetics, and behavioral neurobiology. Moreover, this small teleost is currently being successfully used in other fields, such as pharmacology, and in the discovery and selection of new bioactive compounds or drugs [17,18,19]. The advantages of using zebrafish in biomedical studies are the fact that since these small teleosts share about 70% of their genes with humans, they have conservation of metabolic pathways and neurotransmitter systems similar to those of humans, low maintenance costs, and easy handling [18]. Furthermore, like humans, the zebrafish is a social, diurnal species that relies heavily on vision and reacts strongly to visual information. This fish also performs a repertoire of simple, well-defined, and stereotyped sensorimotor behaviors that have accessible, characterized circuits and provides a large-scale adaptable vertebrate system for neurobehavioral studies [20].

*Coriandrum sativum* L. is a culinary plant of the Apiaceae (Umbelliferae) family, native to the Mediterranean regions and the Middle East [21]. All the vegetative parts of the plant are edible. The leaves are used in cooking as a spice, food preservative, or flavoring agent [22], while the seeds are used in the preparation of various drinks, extracts, or to mask certain specific aromas [23]. Coriander has been widely used since ancient times because of its medicinal and biological properties [24]. In traditional medicine, Coriander is often used as a carminative to treat and relieve diarrhea, dyspepsia, and flatulence or to relieve respiratory or urinary problems. It can also be used as an antiemetic [25]. Recent studies have highlighted the presence of various secondary metabolites in *C. sativum*, with the main constituents being geranyl acetate and linalool [26]; however, CSEO also contains other active compounds such as phenolic acids (gallic, chlorogenic, caffeic, vanillic, p-coumaric, ferulic, rosmarinic, o-coumaric, trans-hydroxycinnamic, salicylic, and trans-cinnamic) and flavonoids (quercetin-3-rhamnoside, rutin trihydrate, luteolin, quercetin dihydrate, resorcinol, kaempferol, naringin, apigenin, flavone, and coumarin) [27]. To date, multiple studies have described this plant as having antidiabetic [28], antimicrobial [29], anti-carcinogenic [30], and antioxidant [27] effects, with the potential to ameliorate Aβ-induced neuronal damage in rodent models of AD [6,31]. The present study explored the mechanism through which CSEO affected anxiety, cognitive performance, and brain antioxidant capacity in the scopolamine (SCOP)-induced zebrafish model.

## 2. Materials and Methods

### 2.1. Experimental Animals and Methods of Work

This study tested 100 wild adult zebrafish (4–6 months) with short fins, at a ratio of 1:1 male–female. The average length of the fish bodies measured between 3 and 4 cm. All fish in the study were procured from an authorized local commercial distributor (Pet Product SRL, Bucharest, Romania). Immediately after procurement, all animals used in the study were placed and observed for two weeks in quarantine. Zebrafish were housed in mixed groups of 10 individuals per aquarium in glass aquaria in a volume of 10 L of dechlorinated tap water, which was replaced every two days. The water was maintained at a temperature of 28.5 ± 1 °C. Water conditions were monitored twice a day in a circulating water system and maintained at the following parameters: pH 7–7.5, dissolved oxygen 8 ± 1 mg/L, conductivity 1500–1600 µS/cm, and at an ammonia and nitrite content of less than 0.001 mg/L, in a 14:10 h cycle (light:dark). Food was given to the animals in equal amounts ad libitum in the form of Norwin Norvital flakes (Norwin, Gadstrup, Denmark) three times a day (early morning-morning − afternoon). The food was available to all fish and was eaten within 10 min. During this period, the air pump was turned off to allow the fish to eat the food. All animal handling in this study minimized potential suffering to the fish by respecting the guidelines of Directive 2010/63/EU of the European Parliament and following research protocols approved by the Animal Research Ethics Committee of the Faculty of Biology, Alexandru Ioan Cuza University in Iasi, Romania (no. 02/30.06.2020).

**Figure 1:**
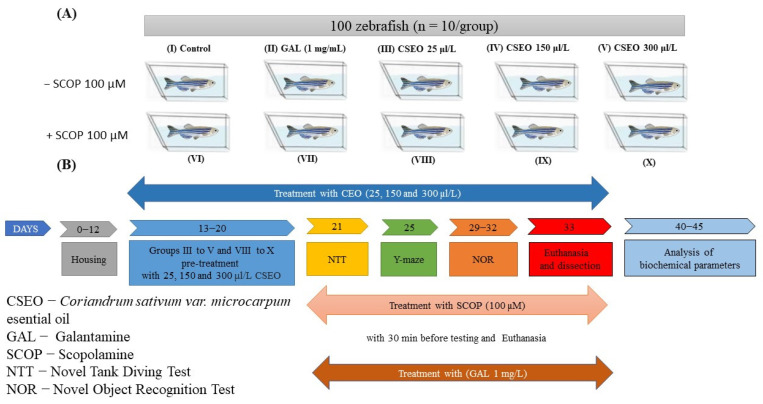
Schematic representation of the experimental design of the study. (**A**) Experimental groups; (**B**) Behavioral and biochemical tests.

The animals were randomly divided into 10 experimental groups (Figure 1A) as follows: (I) control; (II) galantamine (GAL, 1 mg/L) [32], which was used as a positive control in both behavioral and biochemical tests; three treatment groups (III, IV, and V) with *Coriandrum sativum* var. *microcarpum* essential oil (CSEO, 25, 150, and 300 μL/L); (VI) scopolamine (SCOP, 100 μM) [33]; (VII) SCOP (100 µM) + GAL (1 mg/L); and three treatment groups (VIII, IX, and X) SCOP (100 µM) + CSEO (25, 150, and 300 µL/L). Group (II) was acutely treated with GAL (1 mg/L) before testing for 3 min. CSEO was prepared in 1% Tween 80 solution and administered to the zebrafish from experimental groups III–V and VIII–X, respectively, by immersion in water in the housing tanks in concentrations of 25, 150, and 300 µL/L. Treatment doses were selected based on our previously published reports [5,15]. The zebrafish dementia model was induced by treating fish from experimental groups VI–X with SCOP (100 µM) for 30 min before behavioral testing and euthanasia, as previously described in our studies [34,35]. Fish in experimental group VII after SCOP treatment were acutely treated for 3 min before testing with GAL (1 mg/L) (Figure 1B).

### 2.2. Behavioral Analysis

To evaluate the effects of CSEO on the behavior of the tested animals, the activity of the zebrafish was recorded using a digital camera, Logitech HD Webcam C922 Pro Stream, with full HD resolution of 1080 pixels and 30 frames per second (Logitech, Lausanne, Switzerland). Videos were analyzed using ANY maze^®^ software (version 6.3, Stoelting Co., Wood Dale, IL, USA).

### 2.3. Novel Tank Diving Test (NTT)

The novel tank diving test (NTT) examines how zebrafish respond to unfamiliar environments, causing them to initially spend more time at the bottom due to anxiety. However, as they become more familiar with the new setting, they gradually adjust and start exploring more at the top of the tank [36]. Thus, NTT evokes motivational/instinctual conflicts between the geotaxis behavior of diving to the bottom of the “protective” pool and the subsequent vertical exploration of the upper water column, targeting the phenotypic domains of exploration and locomotion [37].

In this study, the evaluation of the effects of CSEO on the anxiety-like state in zebrafish was carried out according to the protocol previously described by Cachat et al. [38]. For this test, we used a glass aquarium with perfectly transparent trapezoidal walls, with a height of 15.1 cm, a length of the base of the aquarium of 23.9 cm, a length of the upper part of 28.9 cm, and a width of 6.1 cm. This aquarium was virtually divided into two equal horizontal sections (upper and lower). During the test period, the tank was filled with 1.5 L of water from the fish’s housing tank and placed on a flat white surface. Each animal was tested individually, once during a 6-min session, during which anxiety-like behavior was measured by recording the time spent in the upper/lower zone of the tank (s), the distance traveled in the top/bottom ratio (m), and the latency to enter the top zone (s). Moreover, as part of the NTT test, the locomotor phenotypes of the zebrafish were evaluated by recording the following parameters: total distance traveled (m), velocity (m/s), and freezing duration (s).

### 2.4. Y Maze

To examine the spatial memory and novelty response of zebrafish, we used the Y-maze test, following the protocol described by Cognato et al. [39], as previously described by Boiangiu et al. [40]. The test tank consisted of a glass aquarium with three equal arms that were 25 cm long, 8 cm wide, and 15 cm high, forming an angle of 12 °C. The three arms of the Y-maze were randomly assigned as follows: (A) the start arm (into which the fish was introduced), (B) the other arm, and (C) the novel arm. Visual guidance landmarks were pasted on the outer walls of each arm of the aquarium. These were in the form of geometric figures (squares, circles, and triangles) made of white paper on a black background. These visual cues were previously established by Cognato et al. [39] given that zebrafish show no signs of preference for or avoidance of these geometric shapes. To facilitate the video analysis, the floor of the aquarium was covered with white plastic, while the remaining areas were covered with black plastic, thus creating a contrast between the fish and the maze. During the experiment, the aquarium was placed on a flat surface and was filled with 3 L of water from the accommodation aquarium to avoid stressing the fish during the experiment.

In this test, each animal was tested individually for 5 min in two separate stages that took place one hour apart. During the first (habituation) phase, each fish was individually introduced to the start arm (A) and allowed to explore only the start arm (A) and the other arm (B) for 5 min. There was no access to the novel arm of the maze (C), which was closed with a transparent glass door. During the second stage, each animal was individually introduced back into the starting arm, but this time, the fish had access to all 3 arms to assess their response to novelty.

In the Y-maze test, locomotor activity was measured by recording several parameters, such as total distance traveled (m) and turning angle (°), while to assess novelty response and spatiotemporal memory, we recorded the time the fish spent in the new arm (C) of the maze.

### 2.5. Novel Object Recognition Test (NOR) 

In this experiment, the NOR test was used to assess the recognition memory of zebrafish using cubes of different colors, following the protocol described by Stefanello et al. [41]. For this test, we used a cube-shaped glass tank with a width of 30 cm and a height of 20 cm. During the experiment, the outer walls of the tank were covered with black canvas to minimize stress on the animals during testing as well as to minimize errors. During the experiment, the tank was placed on a flat white surface and filled with water from the housing tank; the water reached 5 cm from the height of the test tank, covering the objects used during the training session. This facilitated the horizontal swimming of the fish, while reducing the vertical activity of the zebrafish.

The test consisted of two sessions per day for 4 days. More precisely, in the first 3 days, 4 habituation sessions were performed, while on the fourth day, a training session and a test session were performed. Habituation sessions consisted of the zebrafish visually exploring the aquarium during two sessions per day, 5 h apart, for 5 min. The training session took place 12 h apart from the last habituation session and involved exploring two familiar objects for 10 min. Two yellow cubes with a side diameter of 2.5 cm were used as objects during the training session. The cubes were placed in two corners of the aquarium, oriented parallel, at a distance of 5 cm from the nearest wall of the aquarium. After the training session, the animals were subjected to a retention period for 60 min, during which fish in group II were administered GAL, fish in groups VI, VIII, IX, and X were administered SCOP, and fish in group VII were administered both SCOP and GAL.

During the test session, in which each fish was tested for 10 min, one of the previously explored objects was replaced by a novel object (N), which consisted of a blue cube of the same size. Both the colors and the geometric shape of the familiar (F) and N objects were selected based on the results obtained by Faillace et al. [42] and Gaspary et al. [43], who showed that zebrafish show a greater innate preference for cubes and spheres over other geometric shapes. The authors also demonstrated that fish do not show an increased innate preference for yellow or blue colors. Fish recognition memory was assigned as % preference. The preference (%) of the fish for one of the 2 objects (difference between exploration time of N vs. F) was calculated according to the following formula: Exploration time in N/(Exploration time in F + Exploration time in N) × 100. Therefore, fish with better reference memory will remember F and spend more time exploring N.

### 2.6. Preparation of Homogenates and Analysis of Biochemical Parameters

Immediately after the last behavioral test, the animals were placed individually for 10 min in a glass dish containing an ice water immersion (2–4 °C), then rapidly euthanized, as previously reported by Brinza et al. [34]. Whole brains were carefully excised, weighed, and placed in 0.5 mL tubes, and stored at −20 °C until further use.

The next day, the brains of fish from the same batch of animals were weighed individually (~3–6 mg) and homogenized in extraction phosphate buffer solution (0.1 M potassium phosphate buffer solution, pH 7.4 with KCl 1.15%) at a ratio of 1 to 10 using a ball mill (Mikro-Dismembrator U; Sartorius, NY, USA). The homogenate was centrifuged (15 min at 14,000 rpm) and the supernatant was later used for the determination of biochemical parameters.

### 2.7. Determination of Acetylcholinesterase (AChE) Activity

To determine the AChE activity in the homogenates, the photometric method described by Ellman was used [44]. Acetylthiocholine (ATCh) iodide substrate (Sigma, St. Louis, MO, USA) and dithiobisnitrobenzoic acid (DTNB) reagent (Sigma, St. Louis, MO, USA) were added together to a phosphate buffer (pH 7.4). ATCh iodide was hydrolyzed to thiocholine and acetate ions. Then, the enzyme activity was measured spectrophotometrically at 412 nm by monitoring the rate of development of the yellow color produced by the reaction between thiocholine and DTNB. Enzyme activity was expressed in nmoles ATCh/min/mg protein. Protein content was determined in brain samples using the Bradford method [45].

### 2.8. Determination of Superoxide Dismutase (SOD) Activity

In this study, superoxide dismutase (SOD) activity was determined based on the protocol described by Winterbourn et al. [46] and adapted by Artenie et al. [47]. Briefly, we monitored the ability of the enzyme to inhibit the reduction of nitro blue tetrazolium (NBT) by superoxide free radicals, which were generated in the reaction medium by the photoreduction of riboflavin. Samples with the final mixture of 0.067 M potassium phosphate solution, enzyme extract, 0.1 M EDTA solution, 0.12 mM riboflavin solution, and 1.5 mM NBT solution were read at 560 nm. Enzyme activity was related to protein concentration in the extract. Thus, the SOD-specific activity was expressed in enzymatic units per mg of protein determined using the Bradford method [45].

### 2.9. Determination of Catalase (CAT) Activity 

To determine catalase activity (CAT), we used a simple colorimetric method, which was first described by Sinha [48]. Briefly, for each sample, 125 µL of enzyme homogenate and 125 µL of 0.16 M H_2_O_2_ substrate solution were pipetted. After 3 min, the reaction was stopped with 500 µL of potassium dichromate—glacial acetic acid solution, and the tubes were incubated at 95 °C. After 10 min, the tubes were centrifuged at 14,000 rpm for 5 min, and the extinction of the supernatant was read at 570 nm. The activity of the enzyme was expressed in µmoles of H_2_O_2_ consumed/min/mg protein.

### 2.10. Determination of Glutathione Peroxidase (GPx) Activity

In this study, GPx activity was assessed using the protocol described by Fukuzawa and Tokumura [49]. This method is based on the fact that GPx catalyzes the decomposition of H_2_O_2_ with GSH as the reductant, resulting in oxidized glutathione (G-S-S-G) and water. A volume of 78 µL enzymatic extract, 475 µL of sodium phosphate buffer 0.25 M, pH 7.4, 36 µL EDTA solution 25 mM, and 36 µL NaN_3_ solution 0.4 M was added to 1.5 mL tubes. After the samples were incubated for 10 min at 37 °C, 50 µL of 50 mM GSH solution and 36 µL of 50 mM H_2_O_2_ solution were added to each tube and left at 37 °C for 10 min. Immediately after, the reaction was stopped with 730 µL metaphosphoric acid solution 7%, after which the tubes were centrifuged for 10 min at 14,000 rpm. After centrifugation, 100 µL of supernatant was transferred from each reaction tube to new tubes, over which 1270 µL of 0.3 M disodium phosphate solution and 136 µL of 0.04% DTNB solution were added. Exactly 10 min after pipetting the reagents, the samples were read at 412 nm against a mixture of 100 µL distilled water, 1270 µL 3 M Na_2_HPO_2_ solution, and 136 µL 0.04% DTNB solution. Finally, the specific activity of GPx was calculated by relating the enzymatic units (EU) to the protein concentration that was determined using the Bradford method [45].

### 2.11. Determination of the Level of Carbonylated Proteins

To determine the level of carbonylated proteins in the brain of zebrafish, we used a method described by Oliver et al. [50], which consists of the interaction of 2,4-dinitrophenylhydrazine with protein residues in zebrafish brain. This generated 2,4-dinitrophenylhydrazones, which were measured at 370 nm against a mixture of GuHCl and KH_2_PO_4_, as previously described in our study [51]. The results were expressed in nmoles DNPH/mg protein.

### 2.12. Determination of Malondialdehyde (MDA) Level

The determination of the level of peroxidized lipids in the brains of zebrafish was carried out based on the protocol described by Ohkawa et al. [52]. This is based on the interaction of lipid peroxides from animal tissue with thiobarbituric acid, generating a pink color, measured at 532 nm.

### 2.13. Statistical Analysis

Results were expressed as mean ± standard error of the mean (SEM). Differences between means were analyzed using one-way analysis of variance (ANOVA) followed by Tukey’s post hoc multiple comparison test, considering treatment as a factor. Statistical significance was set at *p* < 0.05. Statistical analyzes were performed by GraphPad Prism 9.4 (GraphPad Software, Inc., San Diego, CA, USA). The correlation between behavioral scores, enzyme activities, and lipid peroxidation was estimated by the Pearson correlation coefficient (*r*).

## 3. Results and Discussion

### 3.1. Effects of CSEO on Scopolamine-Induced Anxiety-like Behavior in the Novel Tank Diving Test (NTT)

Anxiety-related disorders are ubiquitous among non-human animals and are associated with multiple neurophenotypes in both rodents and fish [53]. Zebrafish respond to a variety of stressors, such as handling, rapid temperature changes, social isolation, overcrowding, and the environment. These factors can trigger specific abnormal behaviors, such as decreased locomotion or increased time spent in the bottom zone of the water basins by increasing periods and immobility time [54]. Therefore, exposure of zebrafish to novelty evokes a strong anxiety response [55]. The NTT is a reliable, well-documented test widely used in experiments assessing innate behavior in zebrafish that is based on the principle [56].

**Figure 2:**
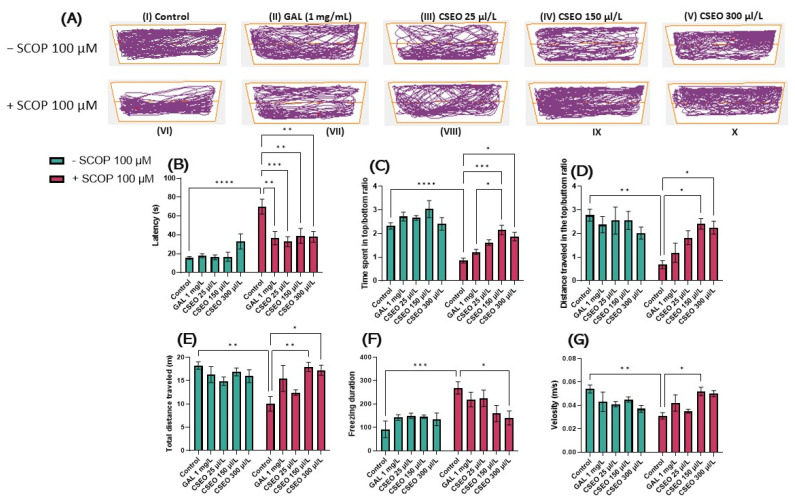
The effects of administering the treatment with *Coriandrum sativum* var. *microcarpum* essential oil (CSEO) in concentrations of 25, 150, and 300 μL/L, both on native zebrafish and those treated with scopolamine (SCOP, 100 µM) in the NTT test. Galantamine (GAL, 1 mg/L) was used as a positive control. (**A**) Graphical representation of zebrafish swimming in the NTT test, with the beginning of the zebrafish’s path represented by the blue dot •, and the end of the fish’s path represented by the red dot •; (**B**) Latency to enter the top zone of the tank (s); (**C**) Time spent in the top/bottom zone ratio; (**D**) The distance traveled in the top/bottom ratio; (**E**) Total distance traveled (m); (**F**) Freezing duration (s); and (**G**) Velocity (m/s) of zebrafish in the novel tank diving test (NTT). Values are expressed as means ± S.E.M., (n = 10 animals per group). For Tukey’s post hoc analyses: * *p* < 0.01, ** *p* < 0.001, *** *p* < 0.0001 and **** *p* < 0.00001.

To evaluate the effects of CSEO(25, 150, and 300 μL/L) on the anxiety-like state in native zebrafish as well as those exposed to treatment with scopolamine (SCOP) (100 µM) in NTT, the following parameters were recorded for 6 min: latency period, time spent in top/bottom area of the aquarium, and the distance traveled in the top/bottom area of the aquarium (Figure 2B–D). To evaluate the neurological phenotypes of the anxiogenic animals, the following behavioral parameters were recorded: total distance traveled, freezing duration, and velocity (Figure 2E–G). In the NTT, throughout a full 6-min session, representative patterns of locomotion tracking highlight the multiple swimming variations of the cohorts of animals tested (Figure 2A). Thus, in the control and galantamine group (GAL, 1 mg/L) fish, there was a tendency to travel a greater distance in the upper zone of the aquarium, highlighting the normal swimming of zebrafish in the NTT test, while in the group of fish treated with SCOP (100 µM), there was an increased preference for the lower area, indicating a high level of fish anxiety and the anxiogenic profile of SCOP. Regarding locomotion tracking patterns in native fish treated with CSEO in concentrations of 25, 150, and 300 μL/L, no major differences were observed compared with the control group. In contrast, fish treated with CSEO in concentrations of 25, 150, and 300 μL/L and subjected to treatment with SCOP (100 µM) showed an increase in locomotor activity compared with the SCOP (100 µM) group, similarly to that of fish in the group with SCOP (100 µM) + GAL (1 mg/L).

Tukey’s post hoc analyses showed that SCOP treatment produced a potent anxiogenic response (*p* < 0.0001). This was exemplified by a significant increase in the latency period required for fish to begin vertical exploration of the test tank (Figure 2B), by significantly reducing (*p* < 0.0001), the time evaluated in seconds (s) spent by fish in the top/bottom ratio (Figure 2C), and by significantly increasing the distance traveled by the fish (*p* < 0.01) in the top/bottom zone of the aquarium compared with the control group (Figure 2D). In addition, treatment with SCOP (100 µM) induced a hypolocomotor effect in zebrafish, manifested by a significant (*p* < 0.01) decrease in the total distance traveled (m) (Figure 2E), a significant (*p* < 0.001) increase in the freezing duration (s) of the fish during the 6-min test (Figure 2F) and the decrease (*p* < 0.01) in the average swimming speed (Figure 2G). These results suggested the degradation of the locomotor activity of the tested animals following the administration of SCOP in a concentration of 100 µM.

The anxiogenic and hypolocomotor properties of SCOP treatment that were observed are in agreement with those previously published by Cognato et al. [39], Sadiki et al. [57], Ioniță et al. [58], and Skalicka-Wozniak et al. [59]; however, its exact psychopharmacological profile remains complex and poorly understood. In Cho et al.’s study [60], treatment with SCOP suppressed the anxiolytic effect of physostigmine, an ACh inhibitor. However, Khan et al. [5] categorized SCOP as a powerful, well-established model for behavioral neurophenotyping, particularly for the induction of anxiety in zebrafish.

As can be seen in Figure 2B, chronic administration of CSEO treatment (25, 150, and 300 μL/L) had a beneficial effect on the latency period (*p* < 0.01) for concentrations of 150 μL/L (*p* < 0.001) and 300 μL/L. Simultaneously, treatment with CSEO in the concentration of 150 μL/L (*p* < 0.05) and in that of 300 μL/L (*p* < 0.001) managed to increase the time fish spent in the top/bottom zone (Figure 2C), the distance traveled in the top/bottom zone of the aquarium (*p* < 0.05) (Figure 2C), the total distance traveled during the test period (*p* < 0.01) for the concentration of 150 μL/L and (*p* < 0.05) for the concentration of 300 μL/L. Moreover, the treatment with CSEO succeeded in the concentration of 300 μL/L (*p* < 0.05) to reduce the freezing duration of the fish. This suggested the beneficial effects of the treatment with CSEO, especially in the concentrations of 150 and 300 μL/ L, on the unconditioned spontaneous behavior of zebrafish by reducing the anxiolytic state and restoring the locomotor deficit induced by SCOP treatment. Regarding the effects of CSEO (25, 150, and 300 μL/L) on the anxiety-like state (Figure 2B–D) and locomotor activity of native zebrafish (Figure 2E–G), no statistically significant effects were observed. However, an increase in time spent in the top/bottom zone was observed in fish treated with CSEO (150 μL/L), as can be seen in Figure 2C.

**Figure 3:**
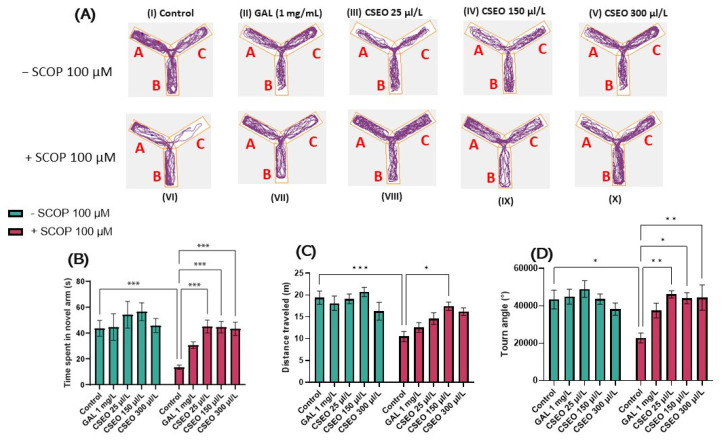
The effects of administering treatment with *Coriandrum sativum* var. *microcarpum* essential oil (CSEO) in concentrations of 25, 150, and 300 μL/L, both on native zebrafish and those treated with scopolamine (SCOP, 100 µM) in a Y-maze. Galantamine (GAL, 1 mg/L) was used as a positive control. (**A**) Graphical representation of the route followed by zebrafish during the second session in the Y-maze. The start of the zebrafish trail is represented by the blue dot •, and the end of the fish path is represented by the red dot •; (**B**) Time spent in the novel arm of the Y-maze (s); (**C**) Distance traveled by fish in the Y-maze during the test session (m); (**D**) Turn angle (°) in the Y-maze. Values are expressed as means ± S.E.M., (n = 10 animals per group). For Tukey’s post hoc analyses: * *p* < 0.01, ** *p* < 0.001, and *** *p* < 0.0001.

As can be seen in (Figure 3C,D), in the Y maze test, the administration of SCOP treatment in a concentration of 100 µM determined a hypo-motor effect, evaluated by the obvious decrease in the total distance traveled (*p* < 0.001) (Figure 3C) and by reducing the turn angle (°) (*p* < 0.05) made by the fish during the test session compared with fish in the control group (Figure 3D). CSEO treatment did not affect locomotor activity in native animals (Figure 3 C,D). In contrast, administration of CSEO treatment at a concentration of 150 μL/L enhanced the total distance traveled by fish, similarly to fish treated with SCOP alone. However, as regards the action of CSEO treatment, a different pattern of action can be observed on the turn angle (°) of the zebrafish in a dose-dependent manner. The most pronounced effect was observed in a concentration of 6 μL/L (*p* < 0.001), but also in concentrations of 3 μL/L (*p* < 0.01) and 1 μL/L (*p* < 0.05).

The results of this study are in agreement with those previously published in the rat model of Alzheimer’s disease (AD) which was induced with beta-amyloid (Aβ, 1–42), where exposure of Aβ (1–42) rats to coriander volatile oil (1% and 3%) significantly increased the time spent in the open arms of the plus maze test, increased swimming time, and decreased immobility time in the forced swimming test [31]. Moreover, administration of the hydroalcoholic extract of *C. sativum* for seven days to a male rat model of seizure induced by intraperitoneal administration of PTZ (100 mg/kg, ip) reduced the duration, frequency, and amplitude of burst discharges while prolonging the latency of convulsive attacks, suggesting the anxiolytic effects of coriander [61]. Furthermore, in a study by Zenki et al. [62], i.p. injection of an extract of coriander leaves at concentrations of 25, 50, and 100 mg/kg into zebrafish ameliorated the anxiogenic effects of the alarm substance.

### 3.2. Effects of CSEO on Zebrafish Spatial Memory Assessed Using the Y-Maze Test

Along with the affective behaviors related to the response to factors associated with anxiety and stress, multiple studies have highlighted the learning abilities [40,63] and memorization [64,65,66] of zebrafish. To evaluate the effect of CSEO on the learning and memory ability of both native and amnesic zebrafish, we used the Y-maze test, following the protocol previously described by Cognato et al. [39]. The principle of this protocol is based on the instinct of zebrafish to show curiosity toward new environments [64]; this task involves several brain regions, such as the hippocampus and the prefrontal cortex. It is therefore a very sensitive test for the deterioration of hippocampal function and the evaluation of drugs with amnestic potential [67].

Figure 3A illustrates the representative tracking patterns of both native, non-SCOP-treated, and SCOP-treated fish on locomotion within the Y-maze and their preference for one of the three arms. It was observed that during the second full 5-min session, the fish in the control group traveled an approximately equal distance in the start arm (A) and the other arm (B), showing a slightly increased preference for the novel arm (C). The group treated with SCOP (100 µM) showed a decrease in locomotor activity and an increased preference for the start arm (A) and other arm (B), exploring to a lesser extent the novel arm (C), suggesting degradation of fish memory following the administration of SCOP treatment. In contrast, both native fish treated with GAL (1 mg/L) and those treated with CSEO (25, 150, and 300 μL/L) as well as those in the experimental groups treated with SCOP (100 μM) + GAL 1 mg/mL and those treated with SCOP (100 µM) CSEO (25, 150, and 300 µL/L) showed an increased preference for the novel arm of the maze.

To evaluate the effects of CSEO (25, 150, and 300 μL/L) on the temporal–spatial memory of both native and SCOP zebrafish in the Y-maze, we recorded the time the fish spent in the novel arm of the Y-maze. In our study, acute 30-min SCOP (100 µM) treatment significantly reduced the time zebrafish spent in the novel arm of the Y-maze (*p* < 0.05), compared with the control fish (Figure 3B). This suggests the amnesic effects of SCOP and impairment of spatial memory in SCOP (100 µM) treated animals. Kim [33] described SCOP as a substance with mixed properties on zebrafish, affecting memory in passive avoidance paradigms. Similarly, in Cognato et al.’s [39] study, the administration of SCOP to zebrafish reduced novel arm exploration in the Y-maze test, suggesting memory impairment in SCOP-treated fish. Moreover, Cakmak et al. [68] and Hong et al. [69] showed that SCOP induces memory loss, especially spatial memory, and general cognitive impairment through cholinergic nervous system dysfunction.

Regarding the effects of CSEO (25, 150, and 300 μL/L) on spatial memory in native fish, it was observed that it failed to enhance in a statistically significant manner the time to explore the novel arm compared with the control group. However, a slight increase in new arm exploration time was observed in fish treated with CSEO at a concentration of 150 μL/L, similarly to that of fish treated with GAL (1 mg/L). However, Tukey’s post hoc analyses showed that in fish from the SCOP (100 µM) treated groups, CSEO treatment was able to increase novel arm exploration time in a significant manner (*p* < 0.05) in all three test concentrations (Figure 3B), highlighting the possible anxiolytic properties of CSEO on SCOP-treated animals.

Our results are in agreement with those previously published by Cioanca et al. [6], in that we showed that coriander volatile oil significantly improved spatial working memory in Y-maze tasks and decreased errors in radial-arm maze tasks in Aβ (1–42)-treated rats. Oral administration of a leaf extract (5, 10, and 15% *w*/*w*) for 45 days to both young and aged rats effectively reversed SCOP-induced memory impairment (0.4 mg/kg, i.p.) in the plus maze and Hebb–Williams models of memory testing [70]. Recently, Mima et al. [71] showed that coriander volatile oil extract could improve the working memory of SAMP8 mice, which are widely used for aging research to study phenotypes such as immune dysfunction, osteoporosis, and brain atrophy. 

### 3.3. Effects of CSEO on Zebrafish Reference Memory Assessed in the NOR Test

**Figure 4:**
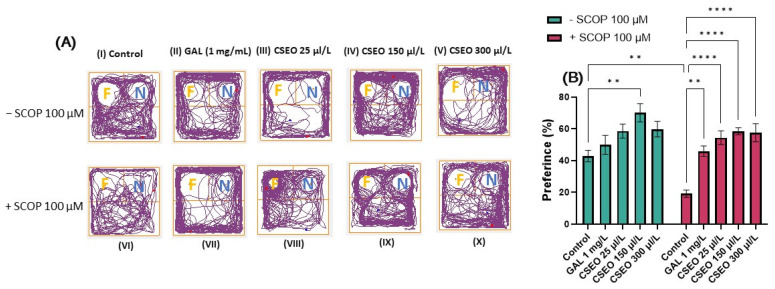
The effects of administering treatment with *Coriandrum sativum* var. *microcarpum* essential oil (CSEO) in concentrations of 25, 150, and 300 μL/L, both on native zebrafish and those treated with scopolamine (SCOP, 100 µM) in the novel object recognition test (NOR). Galantamine (GAL, 1 mg/L) was used as a positive control. (**A**) Graphical representation of zebrafish track during the test session from the NOR test. The beginning of the zebrafish trail is represented by the blue dot • and the end of the fish path is represented by the red dot •; the familiar object area is denoted by the letter (F), while the novel object area is denoted by the letter (N); (**B**) preference (%) for the novel object in the NOR test. Values are expressed as means ± S.E.M., (n = 10 animals per group). For Tukey’s post hoc analyses, ** *p* < 0.001 and **** *p* < 0.00001.

The ability to distinguish previously encountered objects, events, or places is due to reference memory. This ability is crucial for many activities related to daily life and appears very early in the development of several species [72]. To investigate the effects of CSEO on the reference memory of zebrafish, we used the NOR test, which can be used to evaluate the preference of zebrafish for novelty. Zebrafish exhibit visual processing skills and can discriminate colors, lines, and geometric shapes for guidance. Moreover, studies in the literature indicate their ability to differentiate and recognize both real and virtual objects [73]. In this regard, Gaspary et al. [43] showed that zebrafish naturally tend to spend 61% of total test time exploring novel objects (N) over familiar objects (F) and spend 63% of test time exploring the novel location over the familiar one. Therefore, the zebrafish can distinguish a novel object from a familiar object [74]. In the present study, we evaluated the effects of CSEO on the reference memory of both native and SCOP-treated zebrafish using complex geometric objects consisting of yellow and blue cubes. Zebrafish locomotion tracking graphs (Figure 4A) highlight the path taken by the fish during the 10 min of testing. Fish in the control group preferred to explore area N over area F, while fish in the SCOP-treated group showed a preference for F over N.

Tukey’s post hoc analyses revealed that fish treated with SCOP (100 µM) showed a significant decrease in preference percentages (%) (*p* < 0.01) compared with the control group (Figure 4B), suggesting an amnesic effect of SCOP on the recognition memory of zebrafish. The impairment of recognition memory following the administration of SCOP treatment to zebrafish was also previously presented by Boiangiu et al. [75], Brinza et al. [76], and Cognato et al. [39]. Similarly, Yadang et al. [77] stated that the injection of SCOP into Wistar rats induces the appearance of cognitive deficits in the NOR test, directly affecting the learning and recognition process in the rats.

In contrast, the administration of CSEO to native fish increased the percentage of preference for N at a concentration of 150 µL/L (*p* < 0.01) compared with the control (Figure 4B). This indicates the potential beneficial effects of CSEO on recognition memory. At the same time, treatment with CSEO managed to restore the percentage of preference in all three tested concentrations (*p* < 0.0001) to a level such as that of GAL (*p* < 0.01) and in the groups previously subjected to treatment with SCOP, thereby mitigating its negative effects (Figure 4B). The data from our study agree with those in the literature. Thus, multiple studies [71,78,79,80,81] have demonstrated that coriander leaf extract improves recognition memory in an AD rat model by increasing the time to investigate the novel object during the test period.

### 3.4. Effects of CSEO on Acetylcholinesterase (AChE) Activity

The balance of various neurotransmitter systems such as dopamine, acetylcholine (ACh), gamma-aminobutyric acid, noradrenaline, serotonin, and glutamate is indispensable for proper brain function. Cholinergic dysfunction is implicated in the degradation of cognitive processes that occur in adult dementia disorders, including AD [82]. This hypothesis has been supported by the results of multiple neurochemical studies performed on human subjects with AD. These studies revealed low levels of ACh in brain areas that direct cognitive processes in animals, such as the cerebral cortex and the hippocampus [83]. ACh also promotes the transmission of information at the level of the central nervous system (CNS). Therefore, an increase in ACh level is correlated with high memory. Thus, acetylcholinesterase (AChE) inhibitors, able to inhibit the degradation of ACh by AChE, are selected to direct AD progression or improve learning and memory abilities [84]. However, some reports also highlight the negative effects of ACh overexpression on cognitive processes [85]. 

**Figure 5:**
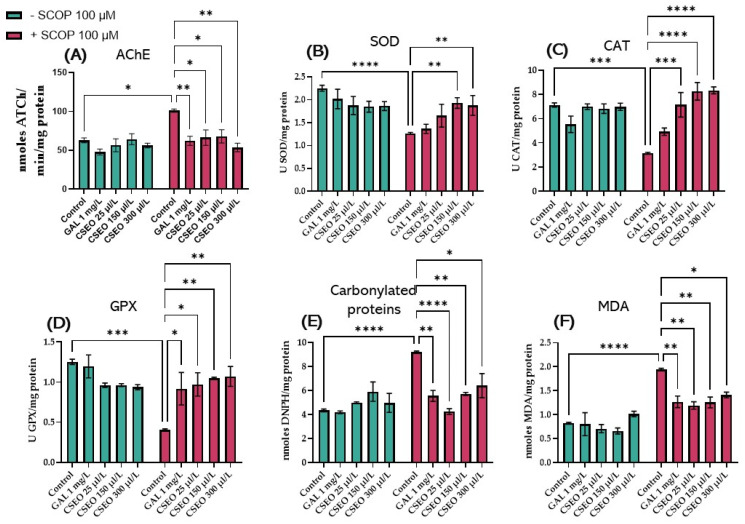
The effects of administering treatment with *Coriandrum sativum* var. *microcarpum* essential oil (CSEO) in concentrations of 25, 150, and 300 μL/L, both on native zebrafish and those treated with SCOP (100 µM) on (**A**) acetylcholinesterase (AChE); (**B**) superoxide dismutase (SOD); (**C**) catalase (CAT); (**D**) glutathione peroxidase (GPx) specific activities; (**E**) carbonylated proteins; and (**F**) malondialdehyde (MDA). Galantamine (GAL, 1 mg/L) was used as a positive control. Values represent means ± SEM (n = 10) followed by Tukey’s post hoc analyses: * *p* < 0.01, ** *p* < 0.001, *** *p* < 0.0001, and **** *p* < 0.00001.

Exposure of zebrafish to SCOP treatment (100 μM) for 30 min amplified AChE activity in the zebrafish brain compared with the control group (*p* < 0.01), which had not been exposed to SCOP treatment (Figure 5A). The rise of AChE activity in the zebrafish brain following the exposure of fish to SCOP has previously been reported in multiple studies aiming to quantify AChE activity [40,86]. Interestingly, native fish in the GAL-treated group (1 mg/L) and native fish in the CSEO-treated groups (25, 150, and 300 μL/L) did not show statistically significant differences in AChE activity, suggesting the role of CSEO and GAL in modulating AChE activity and maintaining it at normal physiological values. However, zebrafish from groups treated with CSEO (25, 150, and 300 μL/L), and then subsequently treated with SCOP (100 μM), were able to decrease AChE activity in the brain in a dose-dependent manner (*p* < 0.01 for the concentration of 25 and 150 μL/L and *p* < 0.001 for the concentration of 300 μL/L). This is in comparison with the group of fish treated only with SCOP. A similar effect was observed in the group of fish treated with SCOP and GAL (Figure 5A). Although in this study, we first demonstrated the anti-AChE effects of CSEO in zebrafish brains, the anti-AChE effects of coriander have also been demonstrated by Hajlaoui et al. [28], who showed in their study that *Carum carvi* and *C. sativum* essential oil, both alone and in combination, show strong anti-AChE activity. In addition, the main constituent of CSEO, Linalool [31], is described in multiple reports as a monoterpene compound with beneficial effects on major brain neurotransmitters such as dopamine, γ-aminobutyric acid (GABA), glutamic acid, and ACh. Moreover, linalool possesses antinociceptive, anti-inflammatory, and antihyperalgesic activities in various animal models [87,88].

### 3.5. Effects of CSEO on Oxidative Stress

Oxidative stress is one of the main pillars that induces the development of AD, characterized by the generation of multiple free radicals in the intracellular space, such as OH, O_2_, H_2_O_2_, HOO, and NO [89]. The continuous and large generation of O_2_ and H_2_O_2_ leads to the development of tissue damage involving the formation of OH radicals, which are known to be highly reactive, as well as other oxidizing molecules in the presence of catalytic iron or copper ions [90]. By stopping the chain reactions of free radicals, catalase (CAT) is involved together with superoxide dismutase (SOD) and glutathione peroxidase (GPx) in the essential antioxidant defense mechanisms of DNA, proteins, and lipids. SOD overexpression has also been experimentally shown to reduce hippocampal HO levels and thereby prevent memory deficits in a mouse model of AD [91].

In this study, the administration of SCOP treatment (100 µM) for 30 min to zebrafish showed a significant decrease in SOD activity (*p* < 0.0001) (Figure 5B), CAT activity (*p* < 0.001) (Figure 5C), GPx activity (*p* < 0.001) (Figure 5D), carbonylated protein level (*p* < 0.0001) (Figure 5E), and malondialdehyde (MDA) level (*p* < 0.0001) (Figure 5F) compared with the control group. Earlier, Wang et al. [92] demonstrated that SCOP damages the cholinergic system and induces the generation of oxidative stress in both mice and zebrafish. Alghamdi et al. [93] showed that SCOP alters the cholinergic status in rat brains by disrupting the activity of AChE and acetyltransferase (ChAT). This occurs by establishing the oxidative status and changing the activities of glutathione (GSH), SOD, and CAT; increasing the levels of interleukin-10 (IL-10); reducing the level of brain-derived neurotrophic factor (BDNF); and increasing the levels of MDA, nitrate, IL-1β, IL-6, and tumor necrosis factor-alpha (TNF-α) in brain tissue.

In our study, Tukey’s post hoc analysis revealed no statistically significant difference between the groups of native zebrafish treated with CSEO (1, 3, and 6 μL/L) versus the control fish regarding the activity of SOD (Figure 5B), CAT (Figure 5C), GPx (Figure 5D), the level of carbonylated proteins (Figure 5E) and the level of MDA (Figure 5F). However, administration of treatment with CSEO (1, 3, and 6 μL/L) to fish that also received treatment with SCOP (100 μM) registered a significant increase in SOD activity at concentrations of 3 and 5 μL/L (*p* < 0.01). This is in comparison with the group treated with SCOP alone (100 μM). However, it was noted that treatment with CSEO at a concentration of 1 μL/L failed to restore the enzyme activity (Figure 5B). Moreover, as can be seen in Figure 5C, in SCOP-treated animals, CSEO treatment was able to positively regulate CAT activity in all three concentrations tested (*p* < 0.001 for the 1 μL concentration/L and *p* < 0.0001 for concentrations of 3 and 5 μL/L). Moreover, Tukey’s post hoc analysis revealed an improvement in GPx activity in fish from SCOP (100 μM) + CSEO (1 μL/L) groups (*p* < 0.05), SCOP (100 μM) + CSEO (3 μL/L) (*p* < 0.01) and SCOP (100 μM) + CSEO (1 μL/L) (*p* < 0.01) compared with the SCOP (100 μM)-treated group (Figure 5D). In addition, CSEO treatment was able to reduce the level of carbonylated proteins in the brain of SCOP-treated zebrafish, especially at a concentration of 1 μL/L (*p* < 0.0001). However, it showed a statistically significant effect in higher concentrations (*p* < 0.01 for the concentration of 3 μL/L and *p* < 0.05 for the concentration of 5 μL/L) compared with fish in the SCOP group (100 μM) (Figure 5E). Chronic administration of CSEO treatment (1, 3, and 6 μL/L) to fish with an amnesia model induced by acute administration of 100 μM SCOP for 30 min significantly restored the antioxidant status in the brain of the tested animals, but a varied pattern of efficacy was observed. Thus, it was found that the treatment with CSEO in concentrations of 1.3 μL/L attenuated the level of lipid peroxidation with the greatest efficiency (*p* < 0.01), in a similar way to that in the group treated with SCOP + GAL (*p* < 0.01). Treatment with CSEO at a concentration of 5 μg/L showed more modest potential on the level of lipid peroxidation (*p* < 0.05), as can be seen in Figure 5F.

These results agree with those obtained by our previous group in the amyloid β (1–42) rat model of AD [6], where treatment with CSEO (1 and 3%) was able to restore the specific activity of SOD and GPx and reduce the level of MDA. Moreover, in another more recent study, CSEO (1 and 3%) restored CAT activity and positively modulated reduced total GSH content in the hippocampus of Aβ (1–42) rats [31]. Our findings regarding the effects of CSEO on SOD, CAT activity, and MDA level are in agreement with the results of Sreelatha et al. [94], who showed that oral administration of coriander stem and coriander leaf at individual doses of 100 and 200 mg/kg to Wistar albino rats with carbon tetrachloride (CCl_4_)-induced liver necrosis and a steatosis model had the effect of protecting the liver of CCl_4_-induced oxidative stress damage by restoring SOD, CAT, and GPx activity. These results are comparable with the standard drug, “Silymarin,” in increasing protective enzymes. Moreover, the polyphenolic compounds in coriander even show a protective effect against H_2_O_2_-induced oxidative stress in human lymphocytes [25,95]. Park et al. [96] showed that *C. sativum* L. extract protects human keratinocytes from oxidative stress by regulating oxidative defense systems. Coriander also protects HaCaT cells by increasing glutathione levels and SOD and CAT activity. Moreover, it increased the expression of activated nuclear factor erythroid 2-related factor 2 (Nrf2), which plays a crucial role in protecting skin cells against oxidative stress [25]. Nishio et al. [97] indicated that the intake of coriander leaf extract contributes to the strong resistance to oxidative stress in the kidneys by decreasing the concentration of heavy metals, especially arsenic. Kajal et al. [98] showed that coriander improves neuronal function by inhibiting oxidative/nitrosative stress and TNF-α in diabetic rats. In addition, Prachayasittikul et al. [99] stated that the antioxidant activity of *C. sativum* generates a protective shield against neuronal oxidative damage, thereby preventing chronic brain inflammation. The authors also stated that by modulating receptors in related CNS pathways, the properties of coriander’s key bioactive compound (linalool) also contribute to the plant’s anticonvulsant, antidepressant, and analgesic properties. Coriander’s antioxidant properties are also attributed to its high levels of phenolic acids, such as protocatechuic acid, caffeic acid, and glycitin, with 2.734 mg of catechin equivalents per 100 g in dry samples [100].

### 3.6. Pearson Correlations between Behavioral and Biochemical Parameters

**Figure 6:**
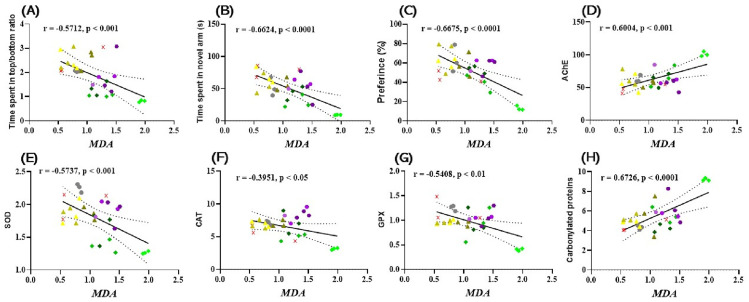
Correlation analyses between behavioral and biochemical parameters (Pearson’s correlation). Data shown are (**A**) time spent by fish in the upper/lower zone of the NTT and MDA test (n = 10, *r* = −0.5712, *p* < 0.001); (**B**) time spent by fish in the novel arm of the Y vs. maze. MDA (n = 10, *r* = −0.6624, *p* < 0.0001); (**C**) preference (%) in NOR over MDA (n = 10, *r* = −0.6675, *p* < 0.0001); (**D**) AChE vs. MDA (n = 10, *r* = 0.6004, *p* < 0.001); (**E**) SOD vs. MDA (n = 10, *r* = −0.5737, *p* < 0.001); (**F**) CAT vs. MDA (n = 10, −0.3951, *p* < 0.05); (**G**) GPx vs. MDA (n = 10, *r* = −0.5408, *p* < 0.05); and (**H**) Carbonylated proteins vs. MDA (n = 10, *r* = 0.6726, (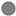) Control, (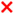) Galantamine (GAL, 1 mg/mL), (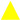) *Coriandrum sativum* var. *microcarpum* essential oil (CSEO, 1 μL/L), (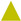) CSEO 3 μL/L, (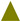) CSEO 6 μL/L, (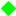) Scopolamine (SCOP, 100 μM), (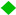) SCOP (100 μM) + Galantamine 1 mg/mL, (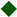) SCOP (100 μM) + CSEO 1 μL/L, (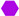) SCOP (100 μM) + CSEO 3 μL/L, (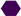) SCOP (100 μM) + CSEO 5 μL/L.

Pearson’s correlation coefficient (*r*) was used to explore the relationship between behavioral scores, enzyme activities, and lipid peroxidation, which included time spent in the top/bottom zone of the aquarium (s), time spent in the novel arm of the Y maze (s), preference (%), AChE, SOD, CAT, GPx, and carbonylated proteins against MDA (Figure 6). Time spent in top/bottom zone (Figure 6A), new arm exploration time (Figure 6B), preference (%) (Figure 6C), SOD (Figure 6E), CAT (Figure 6F), and GPx activity (Figure 6G) showed a statistically significant negative correlation with MDA, with *r* = −05712 (Figure 6A), *r* = −0.6624 (Figure 6B), *r* = −0.6675 (Figure 6C), *r* = −0.5737 (Figure 6E), *r* = −0.3951 (Figure 6F), and *r* = −0.5408 (Figure 6G). In contrast, the ratio between AChE activity (Figure 6D) and the level of carbonylated proteins (Figure 6H) showed a significantly positive correlation with MDA, with *r* = 0.6004 (Figure 6D) and *r* = 0.6726 (Figure 6H).

We previously showed that there is a significant correlation between the decrease in working memory errors, reference memory errors, SOD activity, and MDA level in Winstar Aβ (1–42) laboratory rats that were treated with coriander volatile oil [6]. However, in Aβ (1–42) rats, there was no significant correlation between the behavioral response in the Y-maze and the level of MDA. Recent studies have reported that the establishment of oxidative stress in animal cells occurs as a result of the instability of oxidant and antioxidant factors, and this is one of the main factors leading to the establishment of cognitive imbalances in elderly people. In addition, excess generation of reactive oxygen species induces protein and lipid degradation [101]. Other studies have also demonstrated that there is a significant correlation between the activity of SOD, CAT, and GPx versus the level of MDA in people with different forms of dementia, especially AD [59,60]. In this regard, Bari et al. [102] demonstrated that the evolution of the dysfunction of cognitive processes strongly correlates with the generation and increase of oxidative stress, especially with the levels of MDA in the brain. At the same time, Aykac et al. [103] showed that there is a significant positive correlation between increased AChE activity and increased MDA levels in AD individuals. Therefore, due to its effects on markers of oxidative stress and improvement of behavior in behavioral tests, CSEO could be a potential natural treatment against amnesic and anxiety symptoms.

## 4. Conclusions

The results of this study suggest that CSEO ameliorated cognitive dysfunction and anxiety state, as measured by specific behavioral tasks. Moreover, CSEO exposure reversed SCOP-induced increase of brain oxidative stress by increasing antioxidant enzymes’ specific activity and increasing the levels of protein carbonyl and MDA. Additionally, CSEO inhibited AChE-specific activity, thereby causing cognitive improvement. The findings suggest that memory enhancement occurs through adjustments in the cholinergic system and a decrease in oxidative stress in the brain. Consequently, these results provide proof of CSEO’s potential as a natural and alternative remedy for anxiety and memory loss.

## 5. Limitation and Future Directions

Zebrafish models offer many advantages for studying various biological processes and diseases due to their genetic similarity to humans, optical transparency, and rapid development. However, zebrafish models also have limitations, as they may not fully replicate the complexity of human physiology and behavior. Moreover, the amount of essential oil absorbed by zebrafish is not known, nor is it known whether the observed effects are due to the presence of a compound in high concentration or the synergistic effects between the compounds present in the essential oil. Future research could focus on refining zebrafish models to better represent specific aspects of human biology, including neurological disorders, anxiety, and memory. This could involve developing transgenic zebrafish lines with specific genetic modifications, utilizing advanced imaging techniques to monitor brain activity, and exploring the effects of various compounds or interventions on zebrafish behavior.

## Data Availability

The data presented in this study are available upon request from the corresponding author.
